# Benefits of whole body vibration training in patients hospitalised for COPD exacerbations - a randomized clinical trial

**DOI:** 10.1186/1471-2466-14-60

**Published:** 2014-04-11

**Authors:** Timm Greulich, Christoph Nell, Janine Koepke, Juliane Fechtel, Maja Franke, Bernd Schmeck, Daniel Haid, Sandra Apelt, Silke Filipovic, Klaus Kenn, Sabina Janciauskiene, Claus Vogelmeier, Andreas Rembert Koczulla

**Affiliations:** 1 Department of Medicine, Pulmonary and Critical Care Medicine, University Medical Center Giessen and Marburg, Philipps-University, Member of the German Center for Lung Research (DZL), Marburg 35043, Germany; 2 Hannover Medical School (MHH) Clinic of Pneumology, Hannover, Germany; 3 Physiotherapy Department, University Medical Center Giessen and Marburg, Philipps-University Marburg, Marburg, Germany; 4 Schön Klinik Berchtesgadener Land, Department of Pulmonology, Schönau am Königssee, Germany

**Keywords:** Exercise, COPD exacerbation, Cytokine biology, Pulmonary rehabilitation

## Abstract

**Background:**

Patients with stable COPD show improvements in exercise capacity and muscular function after the application of whole body vibration. We aimed to evaluate whether this modality added to conventional physiotherapy in exacerbated hospitalised COPD patients would be safe and would improve exercise capacity and quality of life.

**Methods:**

49 hospitalised exacerbated COPD patients were randomized (1:1) to undergo physiotherapy alone or physiotherapy with the addition of whole body vibration. The primary endpoint was the between-group difference of the 6-minute walking test (day of discharge – day of admission). Secondary assessments included chair rising test, quality of life, and serum marker analysis.

**Results:**

Whole body vibration did not cause procedure-related adverse events. Compared to physiotherapy alone, it led to significantly stronger improvements in 6-minute walking test (95.55 ± 76.29 m vs. 6.13 ± 81.65 m; p = 0.007) and St. Georges Respiratory Questionnaire (-6.43 ± 14.25 vs. 5.59 ± 19.15, p = 0.049). Whole body vibration increased the expression of the transcription factor peroxisome proliferator receptor gamma coactivator-1-α and serum levels of irisin, while it decreased serum interleukin-8.

**Conclusion:**

Whole body vibration during hospitalised exacerbations did not cause procedure-related adverse events and induced clinically significant benefits regarding exercise capacity and health-related quality of life that were associated with increased serum levels of irisin, a marker of muscle activity.

**Trial registration:**

German Clinical Trials Register DRKS00005979. Registered 17 March 2014.

## Background

Chronic obstructive pulmonary disease (COPD) is a progressive lung disease characterized by irreversible obstruction of the airways. The progression of the disease is associated with recurrent exacerbations that may lead to decline in lung function, quality of life and exercise capacity while increasing the risk for mortality [1]. Cachexia and muscle atrophy [2] are critical extra-pulmonary manifestations of COPD. It is well established that skeletal muscle function (strength and endurance) and structure (fiber quality, capillary density and metabolic capacity) are altered in patients with COPD *i.e.* shifted towards a decreased oxidative capacity of the muscle [3]. In addition, it was shown that the anabolic-catabolic ratio is shifted towards the catabolic state of the muscle, which is accompanied by ischemia-related apoptosis injury [4].

Pulmonary rehabilitation improves exercise performance, dyspnea, and quality of life, reduces the effects of acute exacerbations and prolongs the interval between exacerbations [5,6]. Current guidelines recommend pulmonary rehabilitation for all stages of COPD but do not explicitly recommend it during an acute exacerbation [1]. The central components of rehabilitation are exercise and strength training. It is hypothesized that the expression of the transcription factor peroxisome-proliferator-activated receptor-γ coactivator 1α (PGC1-α) [7-12] is relevant for the reconstitution of body strength by exercise training. PGC1-α stimulates expression of FNDC5, a membrane protein that is cleaved and secreted as a newly identified hormone called Irisin [13]. The upregulation of PGC1-α has been shown to decrease inflammation and increase angiogenesis [14].

Whole body vibration (WBV) is a novel exercise training modality performed on a vibrating platform that moves in sinusoidal oscillations, during which static and dynamic exercises can be performed [6]. A randomized clinical trial has demonstrated greater improvements in a number of exercise tests in the group of COPD patients (GOLD stages III and IV) trained on the WBV platform compared to a group that was conventionally trained [6].

In the past decades research described pathological features of COPD patients which included lung tissue remodeling, fibrosis, pulmonary and systemic inflammation, lung vascular remodeling, and angiogenesis [15,16]. For that reason, we also evaluated putative beneficial effect of standard physiotherapy programme complemented with WBV relative to standard physiotherapy programme by measuring serum markers of angiogenesis (ICAM-1, VEGF and ACE) and apoptosis (gelsolin, soluble Fas Ligand/TNFSF6, soluble Fas (CD95)/TNFRSF6).

We conducted a proof of concept study in the form of a randomized clinical trial, in which we added WBV training to conventional physiotherapy in a group of patients hospitalised for a COPD exacerbation and compared this group to COPD subjects that only underwent conventional physiotherapy. The goal was to evaluate if WBV training is safe and improves exercise capacity and quality of life, and whether putative improvement is associated with measurable changes in circulating levels of irisin, a recently described hormonal marker of muscle activity.

## Methods

### Patients and study design

COPD patients hospitalized due to severe COPD exacerbations at our hospital between November 2010 and July 2012 were asked for their interest and informed voluntary consent to participate in our clinical trials. Pneumonia was ruled out by chest x-ray. All included patients were treated with an intensification of inhaled bronchodilators and a short term course of systemic corticosteroids. Further information regarding baseline characteristics can be found in Table 1. This hypothesis generating trial has the ethical approval of the local ethics committee (University of Marburg, AZ 197/10).

**Table 1:** Baseline characteristics

**Measured parameter**	**Control**	**WBV**	**p-value**
**Gender [M/F]**	12/8	14/6	0.74
**Age [years]**	70.4 ± 10.1	66.4 ± 9.93	0.24
**Height [cm]**	170.3 ± 10.63	168.9 ± 9.28	0.5
**Weight [kg]**	75.15 ± 19.73	79.5 ± 23.48	0.7
**BMI [kg/m**^**2**^**]**	25.75 ± 6.42	27.88 ± 7.87	0.51
**FEV**_**1 **_**[% pred.]**	38.4 ± 17.82	32.71 ± 13.18	0.43
**GOLD stages [I/II/III/IV)**	1/4/7/7	0/1/11/8	0.3
**Current Smokers [%]**	26.23	30	1.00
Mean Packyears*	47,50 ± 35,94	39,64 ± 27,91	0.53
**On ICS before study [%]**	64.29	70.59	1.00
**On OCS before study [%]**	14.29	23.53	0.66
**Antibiotics [yes/no]**	8/12	11/9	0.53

Data are displayed as mean ± standard deviation. There were no statistical differences between both groups. Mann–Whitney-U test was used to compare ordinal variables, categorial variables (gender, GOLD stages and antibiotics) were tested using the Exact Fisher test. BMI: Body mass index; FEV1: Forced exspiratory volume in one second; ICS: inhaled corticosteroids; OCS: Oral corticosteroids.

Within the first 24 hours of hospitalization, 49 COPD patients were randomised to participate in one of two programmes. The randomization was performed by a third party (a statistician from the sleep laboratory of the University of Marburg). A computer generated list was used to produce envelopes that were stored in a locked room. The investigator who wanted to include a patient called the statistician, reported the patient’s identification number and received the allocation to one of both treatment groups.

Group 1 was a standard physiotherapy programme (Control group, n = 20: 5 min mobilisation, 5 min passive movement, and 10 min respiratory exercises), group 2 was the standard programme complemented with sessions on the WBV device (WBV group, n = 20; Galileo™, Novotec Medical, Pforzheim, Germany). Physiotherapy consisted of mobilisation to bedside and stand, respiratory therapy and passive muscle movements. In the WBV group additional training was performed in a supervised manner 3×2 min/day on the vibrating platform. The patient stood with bended knees on the platform according to published recommendations [17]. The side-alternating movements of the device cause muscle contractions on the entire flexor and extensor chain of muscles in the legs and the trunk [6]. Both training programmes started at the day of study inclusion (the same day or the day after the patient`s admission to the hospital). Details on the training procedures can be found in the Additional file 1: Table S1. The physiotherapists that performed bed-side standard physiotherapy and researchers that performed assessments were blinded for treatment allocation of the patient.

### Clinical assessments

On the days of study inclusion and at the day of discharge from the hospital we performed lung function tests according to ATS/ERS standard procedures [18], ultrasound measurement of rectus femoris cross-sectional area (M. rect. fem.) [19], 6-minute walking test (6-MWT) [20], chair rising test (CRT) (time needed for sitting down and standing up 5 times) as described before [6,17], Saint Georges Quality of Life Questionnaire (SGRQ) [21], COPD assessment test (CAT) [22]. Assessors were blinded for the allocation of the patients.

### Laboratory analysis

Serum level of C-reactive protein (CRP), white blood cells (WBC), alpha-1-antitrypsin (AAT), and interleukin-8 (IL-8) were determined at the routine clinical chemistry laboratory directly at hospitalisation and inclusion (baseline) and on the day of discharge. For the quantitative determination of serum irisin concentrations a commercial ELISA kit (Aviscera Bioscience, INC) was used. Serum samples were measured as duplicate in a plate reader following the instructions manual (Tecan infinite® F200pro). The standard range was between 0,082-1280 ng/ml with a sensitivity of 0,1-0,2 ng/ml.

For quantification of serum ICAM-1/CD54, ACE and VEGF *DuoSet ELISA* Development kits (R&D Systems®) were used. Each serum sample was measured as duplicate and the ELISAs were implemented as recommended in the instruction manual. The standard ranges were between 125–8000 pg/ml for ACE and 15.625-2000 pg/ml for ICAM-1/CD54 and VEGF.

The relative quantification of the transcription factor PCG1-α in serum was performed using Western Blot analyses. Each serum sample was separated by 10% SDS-Polyacrylamid-gelelectrophoresis, transferred to PVDF membrane and detected with a primary antibody against PCG1-α (polyclonal IgG antibody coupled to HRP (Antibodies-online, GmbH) produced in goat, dilution 1:500 in TBST (is mixture of Tris-Buffered Saline and Tween 20) supplemented with 5% milk powder, incubation over night at 4°C). Detection of enhanced chemiluminescence was performed after treatment with secondary antibody (Anti-goat IgG, peroxidase conjugated (Sigma Aldrich®) dilution 1:20000 in TBST with 3% milk powder, 1h at room temperature) with *intas SCIENCE IMAGING* ChemoCam system. After development (10 min) relative quantification of individual band volumes was performed using *LabImage 1D,* 1D Gel and Western Blot Analysis Software (BIOTEC FISCHER) with normalization to one reference sera per blot (for representative sample see Additional file 2: Figure S1).

### Statistical analysis

In this proof-of-concept study the main outcome measure was the between-group difference of the 6-minute walking test (day of discharge – day of admission). Data are expressed as mean ± standard deviation unless stated otherwise. For comparing values at admission and discharge within a group, the Wilcoxon matched-pairs signed-ranks test was employed. To determine between group differences a delta was calculated in each group, in which the difference between the input measurements and final measurement was computed. For these deltas, the Mann–Whitney U-test was conducted to test for differences between the groups. Correlation analysis was performed using Spearman’s correlation coefficient. SPSS 20 (IBM GmbH, Ehningen, Germany) and GraphPad 5.0 (GraphPad Software, Inc., La Jolla, USA) were used. A p-value of < 0.05 was defined as significant. Due to missing data on WBV in exacerbated COPD patients no formal power calculation could be performed.

## Results

### Patient demographics

**Figure 1:**
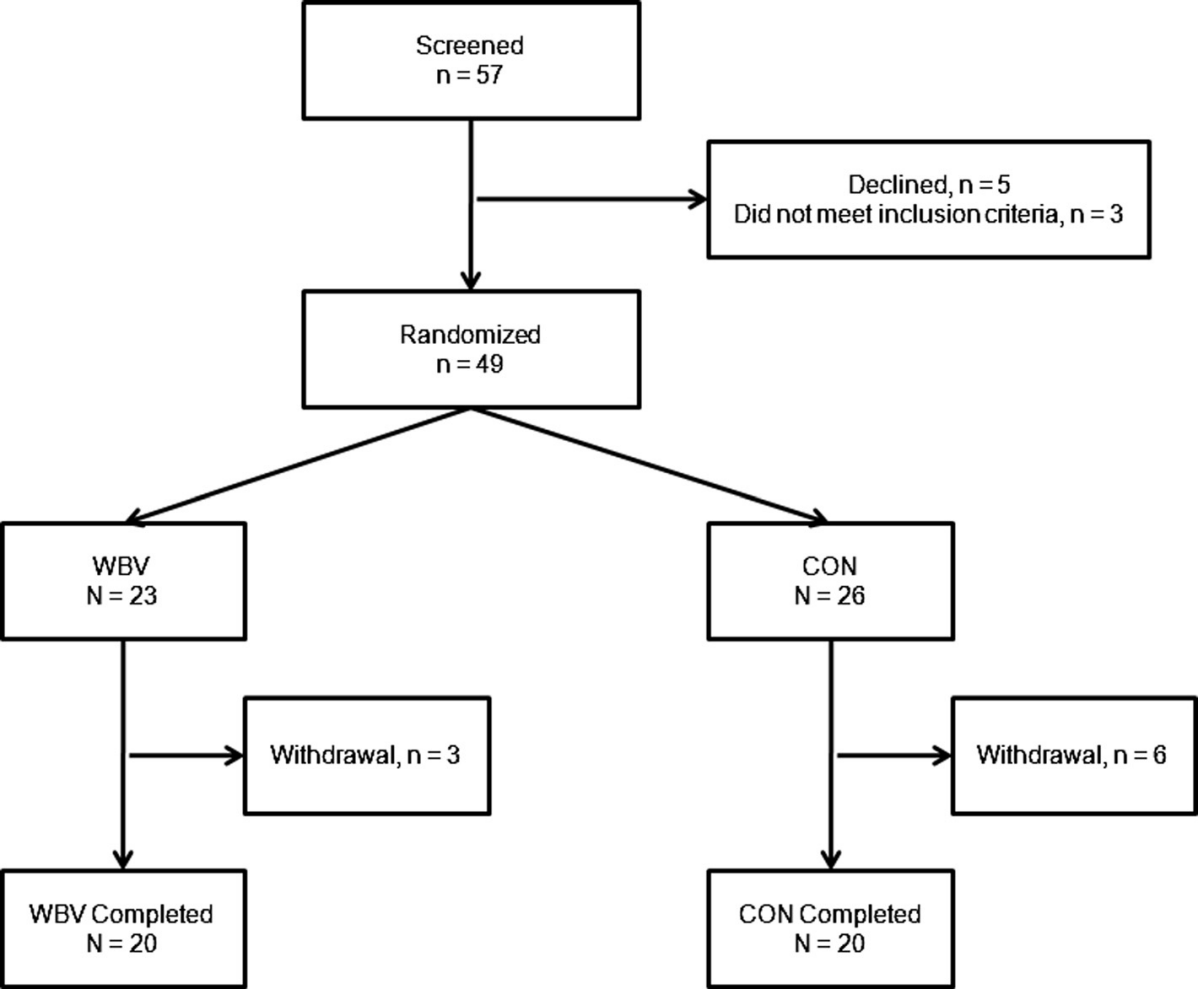
**Trial Profile.** 57 hospitalised patients were screened for randomisation. 3 were not eligible due to pneumonia, 5 patients declined to participate. In the Control (CON) group, 6 patients discontinued training (early discharge: 3; withdrew consent: 2; death: 1). In the whole body vibration (WBV) group, 3 patients discontinued the study (early discharge: 1; withdrew consent: 2).

Between November 2010 to July 2012, 57 patients were screened, 49 were randomized and 40 patients completed the trial (Figure 1). Dropouts were replaced until 20 patients in each group finished the trial. No differential dropout was noted (3/23 vs. 6/26; p = 0.49; Fisher’s exact test). At baseline, there were no significant differences in patient characteristics between the groups. No significant difference in the length of stay could be detected (p = 0.58) (Table 1). A single patient with COPD GOLD 1 was included in the Control group. We analysed it carefully. This patient suffered from comorbidities and had severe symptoms and might display a cluster type which has been described by other groups before (severe symptoms, preserved lung function) [23].

### Lung function

During the time interval between hospital admission and discharge, FEV_1_ increased significantly in both groups (CON: 37.9 ± 17.41% pred. to 43.23 ± 22.8% pred., p = 0.03; WBV: 32.71 ± 13.18% pred. to 36.71 ± 13.89% pred., p = 0.04). Comparing the deltas between both groups no significant difference was detected (p = n.s.).

### Exercise capacity

**Figure 2:**
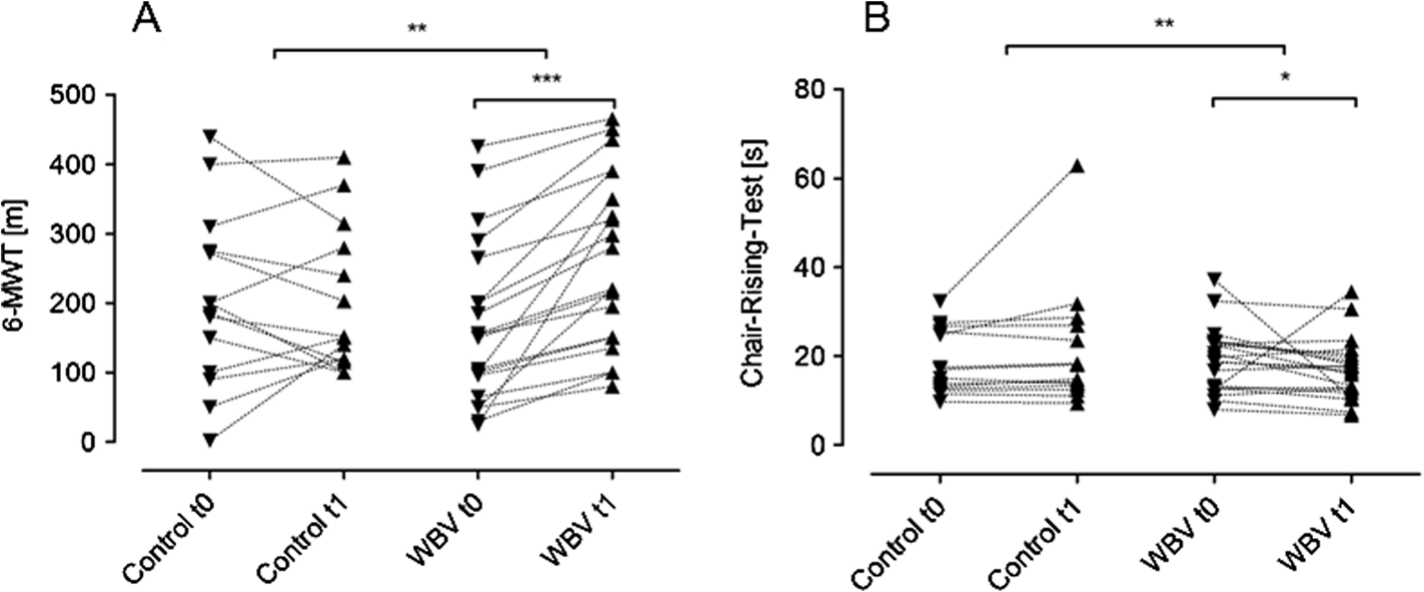
**Exercise capacity and functional testing.** 6-MWT **(A)** and Chair-Rising-Test **(B)**. Whole body vibration (WBV) increased the 6-minute walking test (6-MWT; n = 19) and decreased the time needed for the chair rising test (CRT; n = 14); no significant differences could be detected comparing admission and discharge in the control group (n = 14, n = 14), using Wilcoxon matched-pairs signed-ranks test. When comparing the deltas between both groups we found significantly greater effects in the WBV group. * p < 0.05; ** p < 0.005; *** p < 0.001.

As illustrated in Figure 2A, the 6MWT increased significantly in WBV, but not in the control group (WBV: from 167.9 ± 117.46 m to 263.45 ± 124.13 m; p < 0.001 and CON: from 203.79 ± 126.11 m to 198.67 ± 101.37 m, p = n.s.). The difference between the delta of both groups was significant (CON 6.13 ± 81.65 m vs. WBV 95.55 ± 76.29 m; p = 0.007; Figure 2A).

A similar result was observed for the Chair-Rising-Test (CRT). The time needed for the CRT did not change significantly in CON group (from 18.52 ± 7.32 sec to 28.51 ± 32.05 sec; p = 0.14) but significantly decreased in WBV group (from 19.19 ± 7.43 sec to 17.02 ± 7.04 sec; p = 0.02; Figure 2B). Again, there was a significant difference between the groups comparing the deltas (CON 4.04 ± 9.18 vs. WBV -2.17 ± 8.31; p = 0.003; Figure 2B). There was a negative correlation between the Delta 6MWT and the Delta CRT (r = -0.48; p = 0.04), indicating consistency between both exercise capacity tests.

### Quality of life

**Figure 3:**
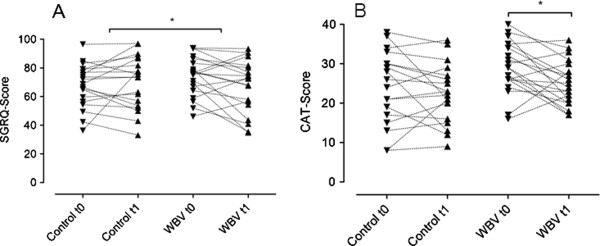
**Quality of Life.** SGRQ **(A)** and CAT **(B)**. Whole body vibration (WBV) had a positive impact on St. Georges Respiratory Questionnaire (SGRQ; n = 20) and COPD Assessment Test (CAT; n = 20); conventional physiotherapy did not influence significantly on SGRQ (n = 19) or CAT (n = 19) (Wilcoxon matched-pairs signed-ranks test). When comparing the deltas between both groups (Mann–Whitney-U test) we found a significant difference in favour of WBV in the SGRQ, but not in the CAT score. * p < 0.05.

As shown in Figure 3, conventional physiotherapy did not change SGRQ (67.61 ± 15.22 to 69.66 ± 18.0) and CAT (24,26 ± 9.14 to 22,65 ± 7.24). In the WBV group, a significant improvement was found regarding CAT (29,05 ± 6.45 to 25,1 ± 5.65; p = 0.02), while SGRQ did not reach statistical significance (74,22 ± 13.84 to 67,79 ± 18.52, p = n.s.). Comparing the deltas between CON and WBV, a significant difference was found only regarding SGRQ (p = 0.049; Figure 3A). Evaluation of the specific domains of the SGRQ, revealed significant group differences only for the activity domain (p = 0.005; Additional file 3: Figure S2). Although the decrease in CAT was more pronounced in the WBV group, the difference between groups was statistically not significant (p = 0.1; Figure 3B). The deltas of SGQR and CAT correlated significantly with each other (r = 0.53; p < 0.001).

### PGC1-α and irisin

**Figure 4:**
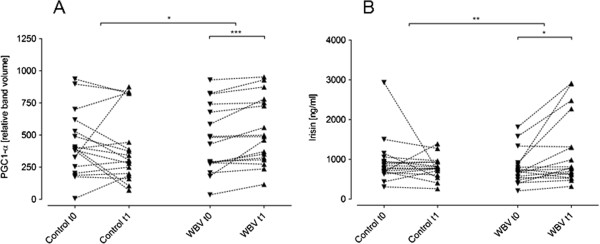
**Markers of muscle activity.** PGC1-α **(A)** and irisin **(B)**. Whole body vibration (WBV) increased peroxisome-proliferator-activated receptor-γ coactivator 1α (PGC1-α) transcription (relative band volume in Western blot analysis; n = 17 for both groups) and serum irisin levels in WBV only. When comparing the deltas between both groups (Mann–Whitney-U test) we found a significant difference in favour of WBV regarding the expression of PGC1-alpha and the expression irisin. * p < 0.05; ** p < 0.005; *** p < 0.001.

Serum PGC1-α levels did not change in the CON group (428.17 ± 249.99 ng/ml to 398.22 ± 272.05 ng/ml, p = n.s.) but significantly increased in the WBV group (460.02 ± 262.28 ng/ml to 529.26 + 260.76 ng/ml; p < 0.001; Figure 4A). Comparing the deltas between both groups, a significant difference was found (CON -29.95 ± 204.08 ng/ml vs. WBV 69.24 ± 75.9 ng/ml; p = 0.02).

Similarly as for PGC1-α, serum levels of irisin did not change significantly in the CON group (934.54 ± 581.98 ng/ml to 791.98 ± 273.83 ng/ml, p = n.s.) but increased in the WBV group (785.96 ± 423.93 ng/ml to 1195.85 ± 875.7 ng/ml; p = 0.01; Figure 4B). Comparing the deltas (discharge – admission) there was a significant difference in favour of the WBV group (CON 142.56 ± 596.26 ng/ml *vs*. WBV 409.89 ± 610.61 ng/ml; p = 0.009).

### Markers of inflammation

On admission to the hospital, both groups had elevated levels of acute phase proteins (CRP, AAT), chemokine (IL-8) and white blood cell counts. The increased levels of WBC counts between the period of admission to discharge in both groups was most likely due to the effects of systemic steroids, which had been given to all patients. CRP, AAT, and IL-8 decreased from admission to discharge (Additional file 4: Figure S3). Comparing the deltas between both groups, the decrease of IL-8 was more pronounced in WBV (Additional file 4: Figure S3; p = 0.04). No other significant inter-group differences could be detected (Additional file 5: Table S2).

### Markers of apoptosis, angiogenesis and remodelling

In both study groups serum markers of angiogenesis (Intercellular adhesion molecule-1 (ICAM-1), vascular endothelial derived growth factor (VEGF), and angiotensin converting enzyme (ACE)) and apoptosis (gelsolin, soluble Fas Ligand/TNFSF6, soluble Fas (CD95)/TNFRSF6) showed changes towards increased vascularisation and decreased apoptosis. However, we found no significant differences regarding these markers between the two groups (Additional file 5: Table S2).

### Adverse events and length of hospital stay

No adverse events were noted that were related to Whole Body Vibration (WBV). There was no difference in the length of hospital stay (CON: 8.63 ± 6.16 days; WBV: 8.58 ± 3.81 days; p = 0.58)

## Discussion

To our knowledge this is the first randomized trial assessing WBV in patients that had been hospitalized because of a COPD exacerbation. We found that WBV improved exercise capacity and quality of life. In addition, there was an increase in serum levels of PGC1-α and irisin, i.e. muscle activity markers that may be induced by the exercise.

A growing body of literature has reported benefit of WBV for patients with cystic fibrosis, [24] multiple sclerosis [25] and stroke [26]. To date, trials regarding efficacy of WBV in patients with COPD are scarce. Results from two studies investigating the effects of WBV therapy in patients with stable COPD showed a significantly greater improvement in the 6MWT, and a significant decrease in maximum oxygen desaturation during the 6-MWT than compared to patients in control group [6,27].

There is a major medical need to improve treatment of patients hospitalized for COPD exacerbations. So far these patients are treated primarily with drugs – bronchodilators, steroids and antibiotics. Recently, studies have been published that evaluate muscle training concepts during exacerbations. Troosters et al. evaluated resistance training and demonstrated improvement of the 6MWD by a median of 34 m after patient discharge [28]. In a small pilot study (n = 15), Abdeallaoui et al. tested neuromuscular electrostimulation and concluded that neuromuscular stimulation is effective in counteracting muscle dysfunction and decreasing oxidative stress [29]. So far, there are no published data analyzing the effects of WBV in patients with COPD exacerbations. With this as a background, we aimed to quantify the clinical benefit of WBV therapy in a group of patients hospitalised for a COPD exacerbation.

We were able to demonstrate that the addition of WBV therapy to a physiotherapy regimen enhances exercise capacity and quality of life. Specifically, we observed a strong effect on 6MWD, which increased by 95.55 ± 76.29 m. The magnitude of the 6WMD improvement was comparable to that described by Pleguezuelos et al. (81.2 m) in the stable phase of the disease [27].

Interestingly, 19/20 patients in WBV group (compared to 9/19 in the control group) improved 6MWD by more than 35 m, which is considered as a minimal clinical important difference (MCID) [30]. Furthermore, 16 out of 20 WBV patients (compared to 12 out of 19 in the control group) displayed improvements in SGRQ of 4 or more units. So far, only one study investigated the beneficial effects of WBV on quality of life in COPD patients and found no difference between WBV and control groups [6]. We guess that the differences between our and previous results could be attributed to the nature of vibration, intensity, and time or amplitude of vibration performance. Due to the paucity of data only preliminary recommendation exist on the practical approach to WBV [17].

When comparing WBV to standard physiotherapy alone (Control) we had to notice that standard physiotherapy only led to very minor improvements. It is well known that the peripheral muscle strength decreases during an hospitalization of COPD [31] Furthermore, a very recent overview states that convincing evidence for the effectiveness of physiotherapy during a hospitalized exacerbation of COPD is missing [32] and the recent BTS guidelines on COPD and pulmonary rehabilitation do not cover that topic [33]. The marginal improvements raise the question on what ground patients were discharged. However, regarding QoL and discharge it has to be acknowledged (Additional file 3: Figure S2) that also patients in the control group improved the symptom subdomain of the SGRQ but did not show an overall improvement. As the decision to discharge a patient is mainly depended on symptoms it seems reasonable why patients had been discharged despite having a worse overall QoL score.

The mechanism of vibration stimulus is not wholly understood; however, it is hypothesized that vibration increases fluid flow, activates muscle spindles, and increases osteogenesis [34]. It is suggested that some of the best-recognized effects of exercise on muscle are mediated by the transcriptional coactivator PGC1-α [14]. PGC1-α is induced in muscle by exercise and stimulates mitochondrial biogenesis, angiogenesis and provides resistance to muscular dystrophy [14]. The benefits of elevated muscle expression of PGC1-α are believed to go beyond the muscle tissue itself. For example, transgenic mice with mildly elevated muscle PGC1-α are resistant to diabetes and have a prolonged life-span [35]. PGC1-α stimulates expression and secretion of hormone irisin, which causes an increase in total body energy expenditure and resistance to obesity-linked insulin-resistance [36]. Hence, irisin reflects benefits of exercise and muscle activity.

We found that clinical improvements in the WBV group paralleled with a marked increase in serum levels of PGC1-α and irisin, the systemic markers linked to muscle physiology. It is important to point out that although the net exercise time was short; we still were able to observe changes in circulating levels of PGC1-α/irisin. To the best of our knowledge, this is the first time that the suggested connection between physical exercise training and the PGC1-α/irisin pathway is supported by the findings from a randomized clinical trial.

When compared to controls, the WBV group also showed a pronounced decrease of serum levels of IL-8 whereas levels of angiogenesis markers, such as ICAM-1, VEGF, and ACE, and apoptosis markers, such as gelsolin, soluble Fas Ligand/TNFSF6, soluble Fas (CD95)/TNFRSF6), did not differ. We cannot say whether the effect of WBV on the IL-8 levels results from a direct modulation of chemokine production by vibration, or if this modulation is secondary to an improvement in muscular properties. Better muscular activity could lower inflammation and result in decreased production of proinflammatory cytokines/chemokines, a possibility that, however, has not yet been properly investigated.

Notably, WBV therapy was well tolerated by the exacerbated COPD patients and no adverse effects were noted during the training program. It was discussed that exercise training during acute exacerbation of COPD may accelerate systemic inflammation [28]. Despite these worries, no increase in serum levels of acute phase proteins, such as CRP and AAT, was found in the WBV training group relative to controls.

This randomized clinical trial has some limitations. First, it was a single centre study, and only 49 patients were randomized. Due to the severity of the disease, especially for the first time point (inclusion), some patients were not able to perform the 6-MWD. To eliminate a potential bias, we calculated the intra-individual effect by setting the admission value to 3 m, which was the lowest values that was obtained by the study. This maximises the effect in the control group. Nevertheless, the inter-group difference in the 6MWD increase was still significant in a favour of WBV (p = 0.009) (Additional file 6: Figure S4). Although the assessment of the SGRQ and CAT did not yield identical results, the deltas of SGQR and CAT correlated significantly with each other (r = 0.53; p < 0.001), demonstrating good agreement. We did not perform a sham procedure; therefore the patients were not blinded for the allocation. Finally, muscle biopsies before and after WBV therapy would provide a major insight in the muscle metabolism, vascularisation and inflammation.

We conclude that WBV is safe, feasible and may exhibit positive effects on clinical parameters (exercise capacity, quality of life) in COPD patients hospitalized due to an exacerbation of their underlying disease. Since the addition of WBV to common exercise training increases the physical activity and enhances circulating levels of the hormone irisin in exacerbated subjects with COPD, it is possible that this training modality within a short timeperiod improves muscle activity, attenuates inflammatory pathways, and improves quality of life. Larger studies are needed to define optimal intensity and duration of WBV as well as to investigate its possible long-term effects.

## Conclusion

Whole body vibration exercise in hospitalised COPD patients did not exhibit adverse events and induced clinically significant benefits regarding exercise capacity and health-related quality of life. The clinical effects of WBV were associated with decreased serum interleukin-8 levels and increased levels of peroxisome-proliferator-activated receptor-γ coactivator 1α (PGC1-α) and irisin, novel markers of muscle activity. This data suggest WBV as a potential training modality during an hospitalized acute exacerbation of COPD.

## Competing interests

The Galileo™ device has been supplied by Novotec Medical, Pforzheim, Germany. No further conflict of interest has to be acknowledged.

## Authors’ contributions

JK, DH, SA, SF, MF, JF performed experiments, measurements and included patients to the study. TG, CV, SJ, CN and KK contributed to the design, statistics and conception of the study, and contributed to drafting the manuscript. ARK contributed to the design and conception of the study. He included patients, analysed and interpreted the data and drafted the manuscript. All authors read and approved the final manuscript.

## Pre-publication history

The pre-publication history for this paper can be accessed here:

http://www.biomedcentral.com/1471-2466/14/60/prepub

## Supplementary Material

Additional file 1: Table S1 Description of Training Programme and Physiotherapy Intervention. COPD patients were randomised to participate either in the standard physiotherapy programme (Control group) or in the standard programme with the addition of exercises on the whole body vibration device (WBV group) Galileo™, Novotec Medical, Pforzheim, Germany). Click here for file

Additional file 2: Figure S1 Western Blot Analysis of PGC1- α. Displayed are three representative blots of peroxisome-proliferator-activated receptor-γ coactivator 1α (PGC1-α) transcript as measured by 10% SDS-Polyacrylamid-gelelectrophoresis. CON: Control; WBV: Whole body vibration. Click here for file

Additional file 3: Figure S2 SGRQ Subdomaines. Displayed are the differences between admission and discharge. When comparing the deltas between both groups (Whole body vibration, WBV: n = 20; Control, CON: n = 19; Mann–Whitney-U test) we found a significant difference in favour of WBV in the activity subgroup of the SGRQ. * p < 0.05. Click here for file

Additional file 4: Figure S3 CRP (a), WBC (b), AAT (c), and IL-8 (d). While white blood cell count (WBC, b) increased (most likely due to systemic steroids), C-reactive protein (a), alpha-1-antitrypsin (c), and interleukin-8 (d) decreased during the course of the study. When comparing the deltas (discharge – admission) between both groups (Mann–Whitney-U test) we found a significant difference in favour of whole body vibration (WBV) regarding the reduction of IL-8. * p < 0.05; *** p < 0.001. Click here for file

Additional file 5: Table S2 Additional Biological Data. Displayed are markers of apoptosis, remodeling and angiogenesis at admission and discharge. Data are displayed as mean ± standard deviation. Wilcoxon matched-pairs signed-ranks test was used to compare differences between day of admission and discharge in both groups and the Mann–Whitney-U-test was used to compare the deltas of the groups (last column). Abbreviations are explained in the text. Click here for file

Additional file 6: Figure S4 6-MWT, Corrections for Missing Values. To account for missing values in the control group, we assumed the 6MWT on the day of admission to be 3 m (lowest measured value). By this we corrected for the underestimation that might have been introduced by missing admission values in the control group. Still, whole body vibration (WBV) increased the 6-minute walking test (n = 19) significantly more than control (CON) physiotherapy (n = 20). * p < 0.05; ** p < 0.005; *** p < 0.001. Click here for file
