# Supplementary figures and images for: Benefits of whole body vibration training in patients hospitalised for COPD exacerbations - a randomized clinical trial
Source: BMC Pulm Med. 2014 Apr 11;14:60. doi: 10.1186/1471-2466-14-60 (PMC4021435; doi:10.1186/1471-2466-14-60)

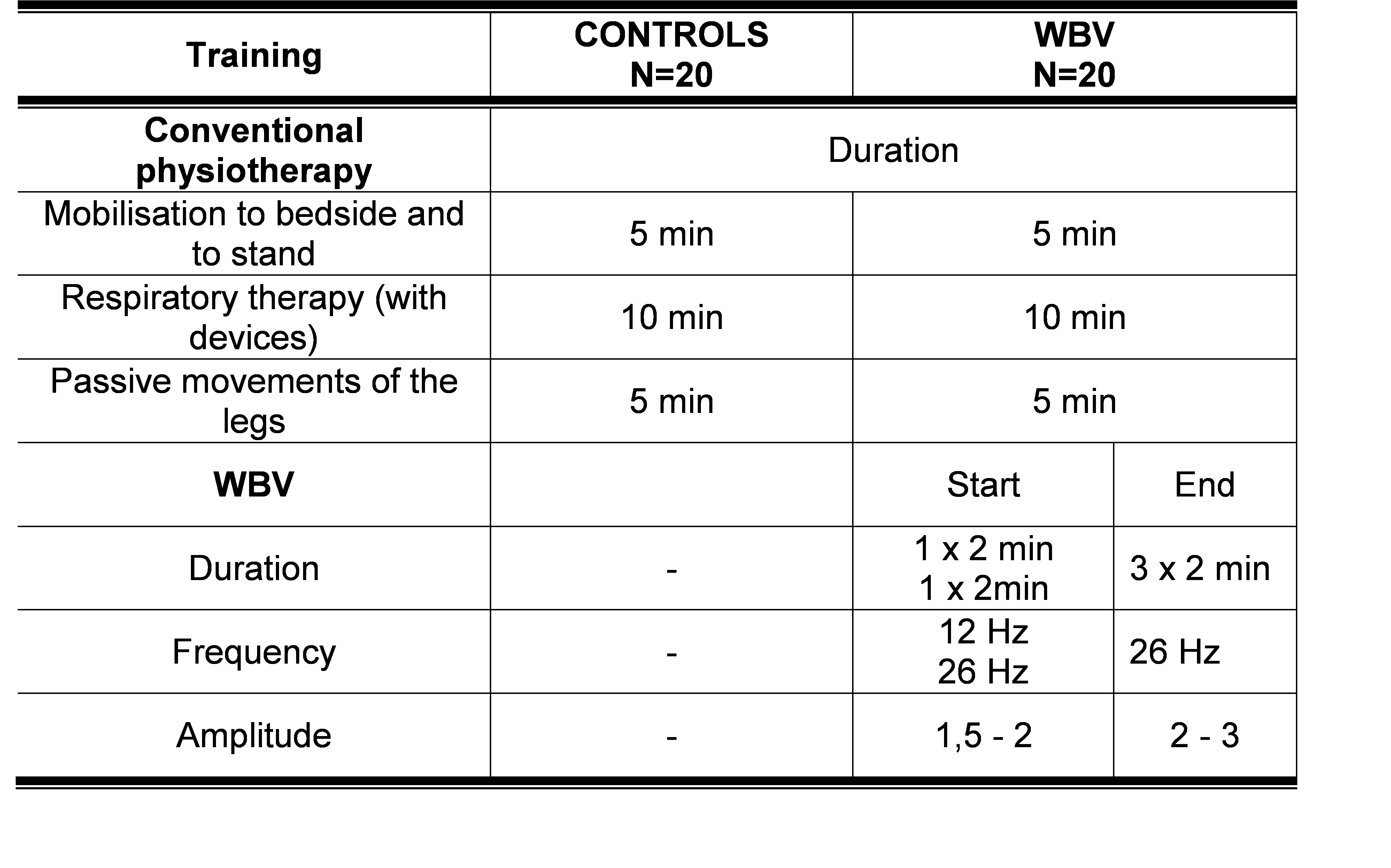

Supplement: Additional file 1: Table S1 — Description of Training Programme and Physiotherapy Intervention. COPD patients were randomised to participate either in the standard physiotherapy programme (Control group) or in the standard programme with the addition of exercises on the whole body vibration device (WBV group) Galileo™, Novotec Medical, Pforzheim, Germany). [file 1471-2466-14-60-S1.tiff]

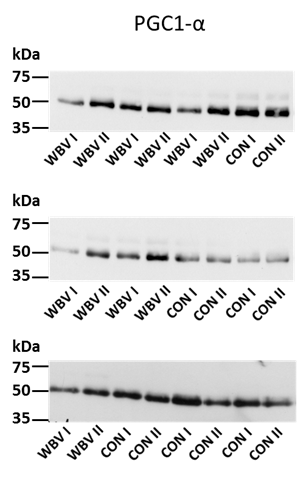

Supplement: Additional file 2: Figure S1 — Western Blot Analysis of PGC1- α. Displayed are three representative blots of peroxisome-proliferator-activated receptor-γ coactivator 1α (PGC1-α) transcript as measured by 10% SDS-Polyacrylamid-gelelectrophoresis. CON: Control; WBV: Whole body vibration. [file 1471-2466-14-60-S2.tiff]

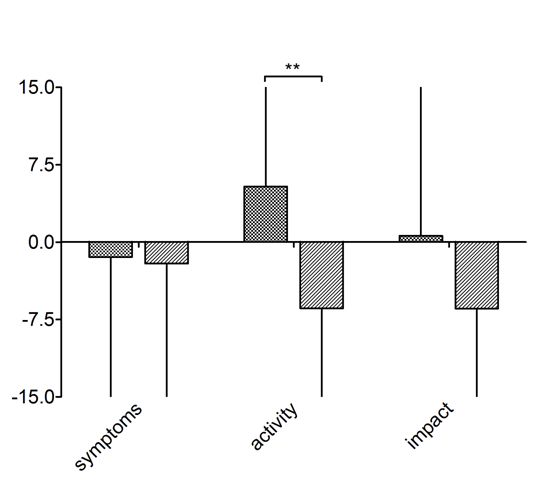

Supplement: Additional file 3: Figure S2 — SGRQ Subdomaines. Displayed are the differences between admission and discharge. When comparing the deltas between both groups (Whole body vibration, WBV: n = 20; Control, CON: n = 19; Mann–Whitney-U test) we found a significant difference in favour of WBV in the activity subgroup of the SGRQ. * p < 0.05. [file 1471-2466-14-60-S3.tiff]

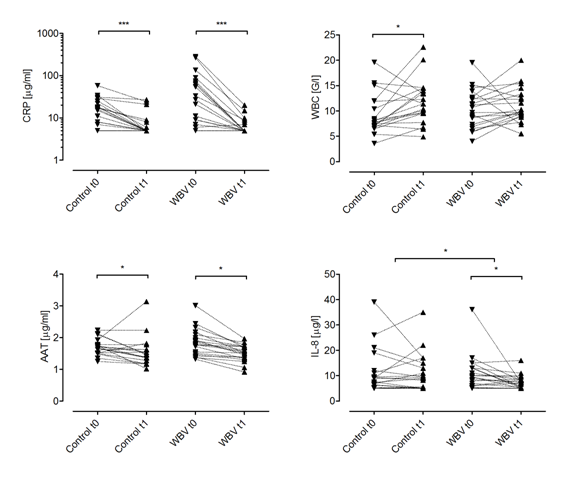

Supplement: Additional file 4: Figure S3 — CRP (a), WBC (b), AAT (c), and IL-8 (d). While white blood cell count (WBC, b) increased (most likely due to systemic steroids), C-reactive protein (a), alpha-1-antitrypsin (c), and interleukin-8 (d) decreased during the course of the study. When comparing the deltas (discharge – admission) between both groups (Mann–Whitney-U test) we found a significant difference in favour of whole body vibration (WBV) regarding the reduction of IL-8. * p < 0.05; *** p < 0.001. [file 1471-2466-14-60-S4.tiff]

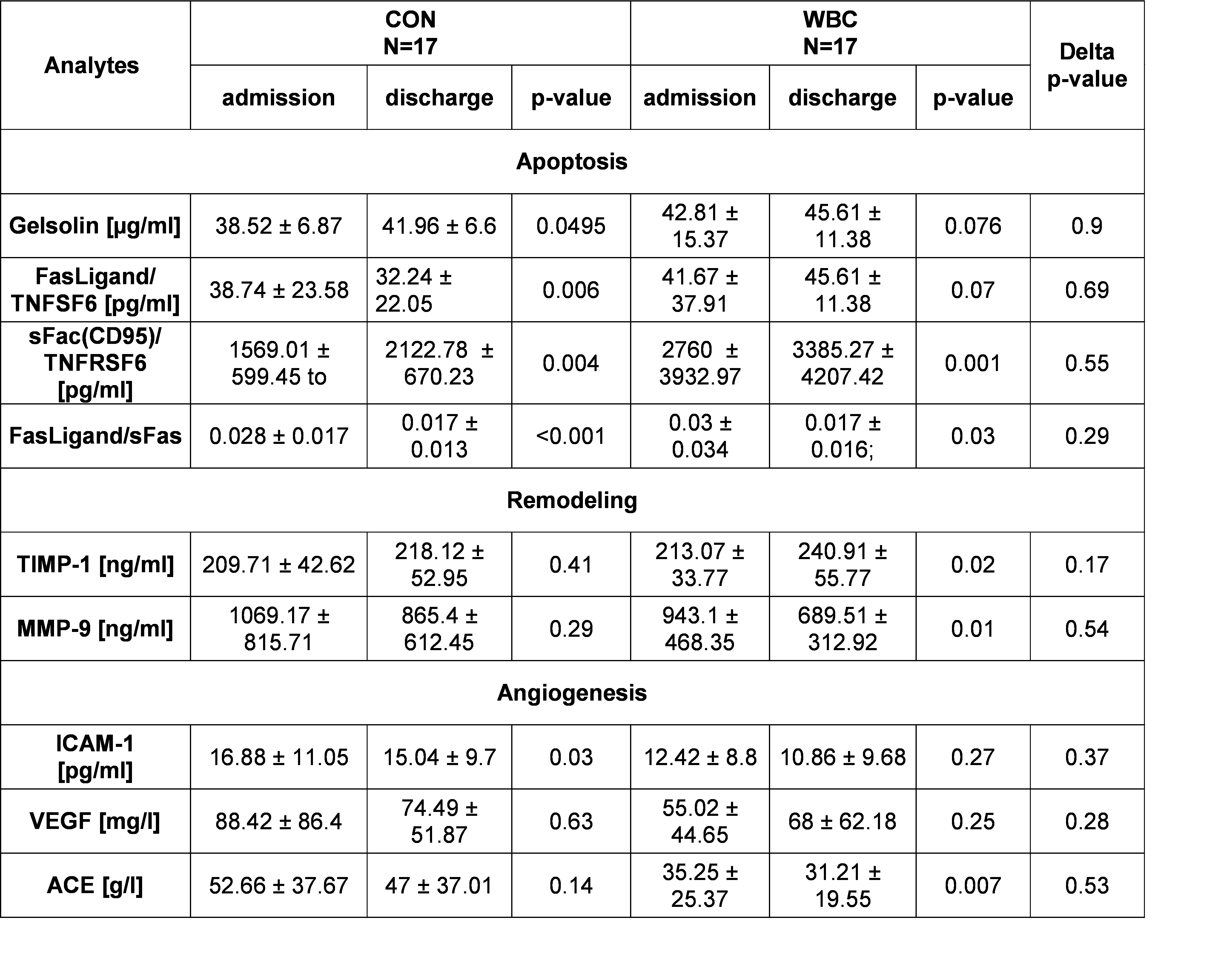

Supplement: Additional file 5: Table S2 — Additional Biological Data. Displayed are markers of apoptosis, remodeling and angiogenesis at admission and discharge. Data are displayed as mean ± standard deviation. Wilcoxon matched-pairs signed-ranks test was used to compare differences between day of admission and discharge in both groups and the Mann–Whitney-U-test was used to compare the deltas of the groups (last column). Abbreviations are explained in the text. [file 1471-2466-14-60-S5.tiff]

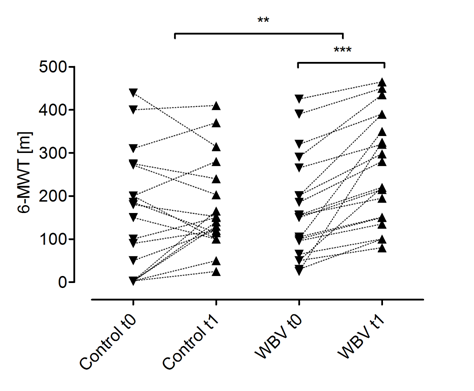

Supplement: Additional file 6: Figure S4 — 6-MWT, Corrections for Missing Values. To account for missing values in the control group, we assumed the 6MWT on the day of admission to be 3 m (lowest measured value). By this we corrected for the underestimation that might have been introduced by missing admission values in the control group. Still, whole body vibration (WBV) increased the 6-minute walking test (n = 19) significantly more than control (CON) physiotherapy (n = 20). * p < 0.05; ** p < 0.005; *** p < 0.001. [file 1471-2466-14-60-S6.tiff]
